# Potential Role of Circulating Tumor Cell Detection and Monitoring in Breast Cancer: A Review of Current Evidence

**DOI:** 10.3389/fonc.2016.00255

**Published:** 2016-12-01

**Authors:** Malgorzata Banys-Paluchowski, Natalia Krawczyk, Tanja Fehm

**Affiliations:** ^1^Department of Gynecology and Obstetrics, Marienkrankenhaus Hamburg, Hamburg, Germany; ^2^Department of Obstetrics and Gynecology, University of Duesseldorf, Duesseldorf, Germany

**Keywords:** breast cancer, disseminated tumor cell, circulating tumor cell, prognosis, biomarkers

## Abstract

The phenomenon of hematogenous tumor cell dissemination in patients with solid tumors has been extensively explored over the last decades. Breast cancer research investigated at first disseminated tumor cells in the bone marrow; however, the focus soon moved to circulating tumor cells (CTCs) in the peripheral blood as blood is easily accessible without an invasive procedure. The prognostic significance of CTC presence has been shown in large studies both in adjuvant and metastatic setting and commercially available detection assays have been evaluated for monitoring in clinical trials. Beyond detection and enumeration of CTCs, the characterization of single tumor cells may enhance our knowledge on disease progression and thus optimize treatment choices.

## Introduction

Breast cancer (BC) is the most common cancer type in women; its mortality is mostly due to distant metastatic growth. The phenomenon of hematogenous spread of single tumor cells shed from the primary tumor was first demonstrated in nineteenth century (1, 2). Tumor cells encountered at secondary homing sites, such as bone marrow (BM) and peripheral blood (PB), are currently seen as surrogate marker for minimal residual disease (MRD) and precursors of distant metastasis. Detection of these cells and evaluation of their features can therefore contribute to our better understanding of the disease and improve therapy monitoring as well as personalized treatment options. The features of tumor cells and changes in the microenvironment at the homing site are the major subject of current translational research.

The prognostic significance of MRD in BC was first demonstrated in studies on disseminated tumor cells (DTCs) in BM. In 2005, a multicenter meta-analysis of BM aspirates collected from 4703 non-metastatic BC patients at time of diagnosis confirmed that DTC presence significantly correlates with shorter disease-free survival (DFS) and overall survival (3). Since venipuncture is more feasible than BM aspiration, subsequent studies shifted their focus to the easily accessible circulating tumor cells (CTCs) in the PB. The following review will address the current knowledge and future clinical possibilities of CTC evaluation in BC.

## Circulating Tumor Cells in Early Breast Cancer

The metastatic cascade consists of a series of steps that enable cells from the primary tumor to enter blood vessels, persist in initially hostile homing sites, and finally extravasate into the tissue of secondary organs. These steps may take place over a prolonged period of time, sometimes decades, involving mechanisms known as tumor cell dormancy (4). Due to improved screening, the majority of BC patients presents with disease localized to the breast and lymph nodes without evidence of distant metastasis. However, after completion of surgical treatment and adequate adjuvant therapy, a significant proportion of patients suffer from a distant recurrence, suggesting that the metastatic cascade had already been activated long before diagnosis.

Accumulating evidence suggests that the fate of tumor cells detached from the primary tumor is highly influenced not only by their own properties but also by microenvironment and immune system. In the blood stream and later in BM and distant organs, these rare cells are exposed to immune response of the host. It is widely accepted that a significant proportion of CTCs dies leaving only a small subpopulation capable of persistence, in a process generally referred to as “metastatic inefficiency” (5). Due to the rapid clearance of CTCs from the blood stream mediated by macrophages, monocytes, neutrophils, and natural killer cells, MRD is under continuous survival pressure to develop mechanisms enabling immune escape (6). Indeed, CTCs have been shown to overexpress proteins inhibiting the phagocytic activity of immune cells and downregulate major histocompatibility class I antigen expression (7–10). It has also been suggested that CTCs are able to stimulate the formation of premetastatic niche in secondary organs. In this context, a number of possible mechanisms have been proposed. Through expression of vascular signal proteins, CTCs can attract VEGFR-expressing hematopoetic stem cells that influence fibroblasts at the homing site, thus creating a favorable microenvironment for future metastatic growth (11). In an animal BC model, tumor cells were shown to induce production of chemokine ligands, especially CCL17 and CCL22, at secondary sites, leading to chemotaxis of both tumor and immune cells. A better knowledge of interactions between CTCs, the immune system and microenvironment could provide us with new therapeutic targets (12).

### Prognostic Significance of CTCs in Early BC

Non-metastatic BC patients in whom tumor cells spread into the blood stream, i.e., who present with CTCs in their PB, are more likely to relapse in the course of disease (Table 1). To date, the largest clinical trial on the prognostic relevance of CTCs in early BC was launched by the German SUCCESS study group (EUDRA-CT No. 2005-000490-21, NCT02181101). Briefly, blood samples from over 2000 average-to-high-risk non-metastatic BC patients before chemotherapy and nearly 1500 patients after chemotherapy were examined (13). Women with detectable CTCs before chemotherapy had significantly worse DFS and overall survival. In this trial, the relationship between CTC numbers and survival was evaluated in order to determine the optimal cutoff (no CTCs vs. ≥1; 0–1 vs. ≥2; 0–4 vs. ≥5 CTCs in 30 ml blood). For all cutoffs, a statistically significant impact on survival was shown. Women with five and more CTCs had highest risk for relapse. So far, the results from the SUCCESS trial were reported after a median follow-up of 36 months. Smaller trials with longer follow-up demonstrated the association between CTC presence and clinical outcome as well (14, 15). In a multicenter pooled analysis, Janni et al. confirmed CTC presence in early BC patients as an independent predictor of shorter disease-free, overall, BC-specific, and distant DFS (16). In the multivariate analysis, for all four survival endpoints alike, grading, tumor stage, nodal stage, hormone receptor status, and HER2 status were additional significant independent prognostic factors, while histologic type, menopausal status, neoadjuvant chemotherapy, and adjuvant chemotherapy were not significantly associated with disease recurrence or survival. Beyond that, the analysis added important new data on the prognostic value of CTCs in various BC subgroups. Most importantly, CTC presence did not significantly influence clinical outcome in low-risk patients with small node-negative tumors, suggesting that early-stage BCs could be treated successfully, independently of the presence of MRD in the blood. In contrast, the strong prognostic value of CTCs in high-risk patients underlines the future potential of CTCs to drive treatment decisions in this patient group. Furthermore, Janni et al. evaluated CTC detection in different molecular subgroups. Hypothetically, tumor cell dissemination patterns may differ according to the biological features of the disease. Indeed, CTC presence predicted survival in patients with tumors of the luminal subtype (i.e., hormone receptor-positive, including luminal B HER2-positive subtype) and with triple-negative tumors, but not in patients with HER2-positive, hormone receptor-negative tumors (16). Contrary to these results, Ignatiadis et al. found in a smaller study no association between CTC presence and prognosis in patients with luminal tumors, while CTC status was highly predictive of survival in the triple-negative and HER2-subtype (17, 18). Similar findings were reported by others (19–21). One aspect that needs to be taken into account is a relatively short follow-up of the available studies; so far, none of the abovementioned trials reported a follow-up longer than 100 months. Especially in case of hormone receptor-positive BC, this might be insufficient to fully assess the clinical relevance of CTC presence as patients with luminal tumors are more at risk for a late relapse compared to those with more aggressive tumor subtypes (22).

**Table 1:** **Presence of CTCs and clinical outcome in non-metastatic BC patients**.

Reference	Patients	Number of patients	CTC positivity	Method	Median follow-up (months)	Association between CTCs and survival
Janni et al. (16) pooled analysis^a^	Stages I–III	3173	641 (20%)	CellSearch	63	DFS, DDFS, BCSS, OS
Rack et al. (13), SUCCESS trial	Stages I–III, node-positive or high-risk node-negative, all pts. received chemotherapy	2026	435 (21%)	CellSearch	36	DFS, DDFS, BCSS, OS
Franken et al. (15)	Stages I–III	404	76 (19%)	CellSearch	48	DDFS, BCSS
Lucci et al. (23)	Stages I–III	302	73 (24%)	CellSearch	35	DFS, OS
Bidard et al. (14), REMAGUS02 trial	Neoadjuvant trial, Stages II and III, ineligible for breast conserving surgery at diagnosis or high-risk	95	22 (23%)	CellSearch	70	DDFS, OS
Molloy et al. (24)	Stages I and II	733	58 (8%)	qRT-PCR (CK19, p1B, EGP-2, PS2, and MmGI)	91	MFS, BCSS
Ignatiadis et al. (17)	Stages I–III, all pts. received adjuvant chemotherapy	444	181 (41%)	RT-PCR (CK19)	54	DFS, OS
Kuniyoshi et al. (25)	Stages I–III	167		RT-PCR (CK19, c-erbB-2)		n.s.
Hwang et al. (19)	Stages I–IIIa	166	37 (22%)	RT-PCR (CK20)	100	MFS, OS

*n.s., not significant; BCSS, breast cancer-specific survival; DDFS, distant disease-free survival; DFS, disease-free survival; OS, overall survival; MFS, metastasis-free survival*.

*^a^Pooled analysis including data from five centers, some previously published as Ref. (13, 14, 15, 23)*.

The ability to assess tumor subtypes has been one of the mile stones toward personalized oncological treatment. In this context, considerable efforts have been undertaken to characterize the phenotype of CTCs and explore potential clinical applications beyond mere enumeration of tumor cells. It has been shown that expression profiles of CTCs do not correlate with the subtype of the corresponding primary tumor (26). However, while the presence of CTCs has been confirmed as an important predictor of worse survival in large trials, evidence regarding prognostic relevance of specific subtypes of CTCs in early BC is scarce. Wulfing et al. assessed the HER2 status of CTCs in a small cohort of 35 stages I–III BC patients and reported a significant association between positive HER2 status and shorter DFS and overall survival (27).

The relationship between DTCs in BM and CTCs in PB has been explored in a number of studies. While both DTCs and CTCs have been shown to predict clinical outcome with level I evidence (3, 16), only a few studies assessed both markers in patients with primary BC. The correlation between CTC and DTC positivity seems weak, ranging from 55 to 76%, depending on the patient population and the method used (28–31). Which “homing site” is better suited for survival prediction remains unclear; while several studies found a stronger correlation between DTC presence and clinical outcome, others provided results in favor of CTCs. The major advantage of blood-based detection is the simplicity of blood sampling in comparison to BM biopsy. Since the procedure is non-invasive, serial measurements are possible.

### CTCs as a Therapy Monitoring Tool in Early BC

Breast cancer patients in whom MRD in the BM persist beyond adjuvant chemotherapy are more likely to be diagnosed with a subsequent relapse (32). Evidence from CTC-based clinical trials showed that persistence of CTCs in the blood is associated with worse clinical outcome as well (Table 2). The SUCCESS trial demonstrated that CTC persistence correlates with shorter recurrence-free and overall survival (13). When both prechemotherapy and postchemotherapy CTC status was considered, the 36-month OS and DFS were higher in patients who were CTC-negative at both time points compared to those CTC-positive before and after chemotherapy (OS: 97.6 vs. 92.8%; DFS: 93.9 vs. 85.9%, respectively). Furthermore, presence of persistent CTCs 2 years after completion of chemotherapy in clinically disease-free patients predicted worse survival as well (33). In this context, one needs to keep in mind that CTCs represent a heterogenous population and that while systemic treatment may eradicate a large proportion of CTCs, tumor cells with enhanced resistant mechanisms may survive and lead to metastatic growth. To date, however, evidence regarding clinical relevance of specific subtypes of persistent CTCs is limited.

**Table 2:** **Presence of persistent CTCs and clinical outcome in non-metastatic BC patients**.

Reference	Patient collective	Number of patients	CTC positivity	Method	Median follow-up (months)	Association between CTCs and survival	Association between CTCs and pathological response of the primary tumor to neoadjuvant therapy
Rack et al. (13), SUCCESS trial	Stages I–III, node-positive or high risk node-negative; blood sample taken after adjuvant chemotherapy	1493	330 (22%)	CellSearch	36	DFS, OS	–
Riethdorf et al. (34), GeparQuattro trial	High-risk non-metastatic BC after neoadjuvant chemotherapy	207	22 (11%)	CellSearch	–	n.d.	No
Kasimir-Bauer et al. (35)	Neoadjuvant trial, Stages II and III, ineligible for breast conserving surgery at diagnosis or high-risk; blood and BM samples taken before and after neoadjuvant chemotherapy	133	11 (8%)	AdnaTest	52	No	No
Hall et al. (36)	Triple-negative early BC after neoadjuvant chemotherapy	57	17 (30%)	CellSearch	30	RFS, OS	No
Bidard et al. (14), Pierga et al. (37) REMAGUS02 trial	Neoadjuvant trial, Stages II and III, ineligible for breast conserving surgery at diagnosis or high-risk; blood sample taken after neoadjuvant chemotherapy	85	15 (18%)	CellSearch	70	No	No

*DFS, disease-free survival; OS, overall survival; RFS, relapse-free survival*.

Furthermore, Hall et al. examined blood samples from 57 patients with triple-negative BC after completion of neoadjuvant therapy and found a significant correlation between CTC presence and shorter relapse-free and overall survival (36). Others reported conflicting results in the neoadjuvant setting (14, 35, 38). Several studies aimed at exploring the interaction between CTC dynamics and pathological changes in the primary tumor during chemotherapy. Pathological complete response has been shown to predict long-term survival and is being used as an endpoint in numerous clinical trials (39). However, changes in CTCs were not associated with tumor’s response to treatment in most studies; in the neoadjuvant REMAGUS02 trial, neither presence of persistent CTCs after chemotherapy nor changes in CTC status correlated with pathological complete response (37). Riethdorf et al. examined blood samples from 213 BC patients before and 207 BC patients after neoadjuvant treatment in the GeparQuattro trial (34). While CTCs could be detected in 22% of patients before start of treatment, the positivity rate decreased to 11% after chemotherapy. However, no correlation between response to therapy and CTC dynamics could be found.

In the neoadjuvant setting, achievement of pathological complete response is significantly linked to favorable survival. In case of adjuvant therapy, this simple tool for response monitoring is no longer available as systemic treatment is administered after surgery. In a study by Xenidis et al., 237 initially CTC-positive patients received either taxane-based or taxane-free adjuvant therapy (40). After a median follow-up of 71 months, patients treated with taxane-based regimen had longer DFS than those receiving taxane-free treatment. Positive effects on survival in the taxane-group were reflected by a shift toward CTC-negative status: 50% of taxane-treated patients turned CTC-negative compared to only 33% in the taxane-free arm.

### Therapy Choices Based on CTCs in Early BC

Treatment decisions in non-metastatic BC are based on the characteristics of the primary tumor without considering features of MRD, although the latter is the aim of any adjuvant strategy and molecular profile of MRD may differ from the primary tumor (41). For instance, we previously reported that 71% of patients with ER-positive BC present with ER-negative DTCs in BM; the loss of hormone receptor positivity may contribute to development of endocrine resistance (42). With regard to another predictive marker, HER2, Riethdorf et al. demonstrated that in 19% of patients with HER2-negative BC HER2-positive CTCs may be detected in PB (34). Similar results were reported with respect to DTCs in BM as well (26, 34, 43). According to current guidelines, patients with HER2-positive MRD but HER2-negative tumors are not eligible for anti-HER2-targeted treatment since only histologically proven HER2 status – either in the primary tumor or in a metastatic lesion – is taken into account. Whether this undertreatment results in worse survival and, consequently, whether these patients benefit from HER2-targeted therapy, remains to be clarified. Rack et al. showed that secondary adjuvant administration of trastuzumab eliminates HER2-positive DTCs from the BM of patients with HER2-negative BC (44). The ongoing TREAT CTC trial (NCT01548677) is the first liquid biopsy-based large trial evaluating the concept of targeting chemoresistant MRD (45). Patients with CTCs persisting beyond (neo)adjuvant chemotherapy are randomized between six cycles of trastuzumab intravenously every 3 weeks vs. observation. In this trial, HER2 status of the CTCs will be assessed, but the treatment is based on the presence of CTCs and not on their HER2 status. Apart from the patients’ characteristics of the pilot phase, this trial has not reported any results yet.

## Circulating Tumor Cells in Metastatic Breast Cancer

### Prognostic Significance of CTCs in Metastatic BC

A total of 40–80% of patients with metastatic BC present with CTCs in PB (Table 3). As demonstrated by Cristofanilli et al., CTC levels above the cutoff value of ≥5 cells/7.5 ml blood at the time of diagnosis are associated with impaired clinical outcome (46, 47). The prognostic value of the threshold of ≥5 CTCs/7.5 ml PB has been further validated by several studies and remains unchanged during the follow-up (37, 47–52). A recent pooled analysis on 1944 metastatic BC patients demonstrated the influence of CTCs on progression-free and overall survival with the highest level of evidence (53).

**Table 3:** **Prognostic value of CTCs in metastatic breast cancer patients**.

Reference	Number of patients	Method	CTC positivity	Association between CTCs and survival
Bidard et al. (53)	1944	CellSearch	47%^a^	PFS, OS
Smerage et al. (54)	564	CellSearch	51%^a^	PFS, OS
Wallwiener et al. (52)	486	CellSearch	42%	PFS, OS
Giordano et al. (51)	517	CellSearch	40%^a^	PFS, OS
Pierga et al. (55)	267	CellSearch	44%^a^	PFS, OS
Müller et al. (56)	254	CellSearch AdnaTest	CSS: 50%^a^ AT: 40%	CellSearch: OS AdnaTest: n.s.
Giuliano et al. (50)	235	CellSearch	40%^a^	PFS, OS
Nakamura et al. (57)	107	CellSearch	37%^a^	PFS
Liu et al. (58)	74	CellSearch	n.s.	PFS
Tewes et al. (59)	42	AdnaTest	52%	OS
Bidard et al. (60)	37	Immunocytochemistry	41%	OS
Nole et al. (61)	80	CellSearch	61%	PFS
Hayes et al. (49)	177	CellSearch	54%	PFS, OS^b^
Budd et al. (48)	138	CellSearch	43%	OS
Cristofanilli et al. (47)	177	CellSearch	49%	PFS, OS

*PFS, progression-free survival; OS, overall survival*.

*^a^≥5 CTCs*.

*^b^At any time during palliative treatment*.

Next to the prognostic role of CTC status, changes in CTC counts in course of treatment have been shown to reflect therapy response: in the analysis by Hayes et al., a decrease in CTC levels under the threshold of five cells in 7.5 ml PB predicted better PFS and OS (49). Furthermore, treatment efficacy assessed by CTC evaluation might be more suitable for therapy monitoring than standard radiological imaging (48). In a prospective trial by Budd et al., CTC persistence in metastatic BC patients predicted impaired clinical outcome despite radiological therapy response (48). In this context, a simple and non-invasive blood analysis for CTCs as a “liquid biopsy” represents an attractive tool allowing a real-time monitoring of disease progression and therapy response.

Beyond CTC enumeration and monitoring of CTC levels during the therapy, characterization of these cells, especially with regard to hormone and HER2 status, has been addressed in several studies on metastatic BC. According to numerous trials, the phenotype and genotype of primary tumor, metastatic lesion, and CTCs often differ (62–66). Hypothetically, CTCs represent the dominant tumor cell population in metastatic disease; therefore, their expression profile may predict therapeutic response most adequately (67). As reported to date, targeted therapy guided by phenotype of CTCs is able to eliminate persistent tumor cells from PB and/or BM of BC patients (44, 68, 69). While prognostic relevance of CTC enumeration in the metastatic setting has been proven in large clinical trials, studies investigating the impact of specific phenotypes of CTCs on survival have yielded contradictory results. Wallwiener et al. evaluated HER2 status on CTCs in 107 metastatic BC patients starting a new line of therapy (65). The HER2 status of CTCs did not influence overall survival. However, patients with HER2-positive CTCs had significantly longer PFS than those with HER2-negative CTCs. In contrast, Hayashi et al. reported worse survival in patients with HER2-positive CTCs (70), and Beije et al. assessed ER and HER2 status on CTCs from 154 MBC patients and reported that none correlated with clinical outcome (71).

Clinical significance of CTC phenotype (in particular, the HER2 status) for guiding treatment decisions and evaluating therapy response in metastatic BC is being currently investigated within the German DETECT trials (NCT01619111). Moreover, the possibility to provide an analysis of CTCs in metastatic BC on a DNA, RNA, and protein level including next-generation sequencing has been demonstrated by recent research (72, 73). CTC characterization on the molecular level might help to identify resistance mechanisms of tumor cells: a crucial step for the optimization of systemic treatment (67).

### CTCs as a Therapy Monitoring Tool in Metastatic BC

While the prognostic relevance of CTC detection has been proven in large clinical trials, its clinical utility remains to be demonstrated (13, 47). Since CTC detection predicts impaired clinical outcome and CTC dynamics seem to reflect treatment response, a question has been raised, whether MBC patients can benefit from CTC-guided therapy decisions. The first large clinical trial to address this issue is the SWOG S0500 study (NCT00382018). In this randomized Phase III trial, metastatic BC patients with persistent high levels of CTCs after first cycle of initial chemotherapy (≥5/7.5 ml of blood) were randomized to switch the therapy or to maintain the current treatment until the clinical evidence of progression (54). While the strong prognostic power of CTCs has been confirmed by this study, treatment change based on CTC persistence did not improve progression-free survival or overall survival in these patients. The clinical outcome was poorest in patients with persistently elevated CTC levels, and these patients might represent a chemoresistant population that requires alternative treatment approaches (54). Furthermore, a currently ongoing study on therapy guidance based on CTC dynamics in metastatic BC is the CirCe01 by Institut Curie, France (NCT01349842). In this multicentre randomized Phase III study, therapy response in CTC-positive patients with disease progression after two lines of chemotherapy is being assessed by clinical tests and radiological imaging or by CTC enumeration. Patients without a significant decrease in CTC levels after first cycle of new chemotherapy will be switched to an alternative regime, which will also be evaluated by CTCs. First results of CirCe01 are expected in 2018 (74). Both studies attempt to demonstrate that patients with persistently elevated CTCs under cytotoxic treatment should be switched off this regimen early in order to avoid inefficient and toxic chemotherapies.

### Therapy Choices Based on CTCs in Metastatic BC

The question whether the choice of systemic treatment in hormone receptor-positive HER2-negative metastatic BC patients might be driven by CTC levels has been raised by the STIC-CTC trial (NCT01710605, Institut Curie, France). In this randomized Phase III trial, the treatment decision will be left at the discretion of the clinicians or according to the number of CTCs in PB: endocrine therapy in case of a CTC count <5 CTCs/7.5 ml PB or chemotherapy in case of a CTC count ≥5/7.5 ml PB.

The worldwide largest trial for therapy guidance according to the CTC status and/or CTC phenotype in metastatic BC patients is the DETECT study concept. In this multicenter study, women with HER2-negative metastatic disease and at least one HER2-positive CTC are enrolled in the DETECT III trial (NCT01619111), and women with HER2-negative BC (hormone receptor-positive or triple-negative) and exclusively HER2-negative CTCs are eligible for the DETECT IV trial (NCT02035813). The recently initiated DETECT V/CHEVENDO trial (Chemo vs. Endo, NCT02344472) completes the DETECT study program with a clinical trial for HER2-positive hormone receptor-positive metastatic BC patients. In the DETECT III trial, patients are randomized to standard systemic treatment of physician’s choice ± additional therapy with lapatinib. In the DETECT IV trial, patients with hormone receptor-positive tumors are treated with endocrine therapy plus everolimus, as a study medication, whereas patients with hormone receptor-positive tumors and indication for chemotherapy as well as patients with triple-negative tumors are treated with eribulin. Metastatic BC patients with HER2-positive hormone receptor-positive tumors are treated with dual HER2-targeted therapy (pertuzumab/trastuzumab) either in combination with chemotherapy or endocrine therapy within the DETECT V/CHEVENDO trial. In DETECT III and DETECT IV, presence of CTCs is mandatory for study inclusion and changes in CTC levels during the treatment are evaluated by repeated blood sampling during the therapy. The accompanying translational research projects of all DETECT studies try to generate additional knowledge of CTCs, their biology and their role in predicting treatment response using methods like single cell analysis, SNaP-Shot technology and next-generation sequencing. This may help to identify new treatment targets and provide more individualized therapy and finally further clarify the clinical value of CTCs in metastatic BC. Currently, ongoing trials on therapeutic utility of CTCs in metastatic BC are summarized in Table 4.

**Table 4:** **Current studies on therapeutic utility of CTCs in metastatic breast cancer**.

Trial	Status	Condition	Intervention	Primary endpoint
SWOG S0500 *NCT00382018* (Phase III)	Active, not recruiting	CTC persistence under chemotherapy	Treatment choice based on clinical and radiological criteria vs. CTC-guided treatment choice	OS
CirCe01 *NCT01349842* (Phase III)	Recruiting	CTC persistence under chemotherapy	Treatment choice based on clinical and radiological criteria vs. CTC-guided treatment choice	OS
STIC-CTC *NCT01710605* (Phase III)	Recruiting	HR+/HER2− MBC	Clinicians choice vs. CTC-guided choice between chemotherapy and endocrine therapy	PFS
DETECT III *NCT01619111* (Phase III)	Recruiting	HER2-negative metastatic BC with HER2-positive CTCs	Standard therapy ± lapatinib	CTC clearance
DETECT IV *NCT02035813* (Phase II)	Recruiting	HER2-negative metastatic BC with HER2-negative CTCs	Endocrine therapy + everolimus (DETECT IV a) or eribulin (DETECT IV b)	PFS
*NCT01975142* (Phase II)	Recruiting	HER2-negative metastatic BC with HER2-positive CTCs	T-DM1	Tumor response rate

## Conclusion

Evaluation of CTCs is one of the most promising biomarkers in solid tumors. In BC, presence of CTCs is an independent predictor of poor clinical outcome in both early and metastatic setting. Numerous potential clinical applications have been proposed and are currently being investigated (Table 5). In case of early BC, the assessment of therapeutic targets is so far restricted to the primary tumor despite increasing evidence of significant discordances between primary tumor and MRD, especially with respect to hormone receptor and HER2 status. Since CTCs might reflect certain, hypothetically most aggressive, subpopulations of the tumor, molecular analysis of these cells and detection of their persistence might identify patients in need of additional or targeted treatment.

**Table 5:** **Potential future applications of CTC detection and characterization**.

Early BC	Metastatic BC
CTC detection might improve prognostication and help to identify patients in need of aggressive therapy and/or bisphosphonates CTC persistence might serve as stratifying parameter to select patients who benefit most from extended endocrine treatment Patients with CTC persistence beyond adjuvant chemotherapy might potentially benefit from secondary adjuvant treatment Evaluation of predictive markers on CTCs might serve as basis for treatment decisions: e.g., patients with HER2-negative primary tumor but HER2-positive CTCs might benefit from HER2-targeted therapy	Detection of high CTC levels and thus worse prognosis might become a valuable information for improved care planning in palliative setting CTC detection after start of a new line of chemotherapy helps to predict response to treatment early; patients with high CTC levels might either be switched to another therapy approach (benefit so far not confirmed in trials) or to best supportive care to avoid unnecessary toxicity Evaluation of CTCs may serve as a liquid biopsy and thus render invasive biopsy of metastasis unnecessary; serial CTC measurements might provide continuous insight into current status of the disease

In metastatic disease, one of the most exciting possibilities is the concept of CTC-guided treatment. Two settings are currently under investigation: first, since several studies have shown that persistently high CTC levels under systemic therapy reflect disease progression sooner than imaging-based monitoring, CTC monitoring might help to identify patients who do not benefit from cytotoxic treatment. However, since an early switch to another chemotherapy regimen in case of high CTC levels after the first cycle of palliative treatment has not shown any survival benefit, it remains to be clarified which therapy options should be favored in such case. Second, a number of clinical trials aim at clarifying whether metastasized patients benefit from targeted therapy based on the molecular profile of CTCs rather than that of the primary tumor.

## Author Contributions

MB-P, NK, and TF contributed significantly to this manuscript. All the authors read and approved the manuscript.

## Conflict of Interest Statement

The authors declare that the research was conducted in the absence of any commercial or financial relationships that could be construed as a potential conflict of interest.

## Abbreviations

BC, breast cancer; BCSS, breast cancer-specific survival; CTC, circulating tumor cell; DDFS, distant disease-free survival; DFS, disease-free survival; DMFS, distant metastasis-free survival; DTC, disseminated tumor cell; EpCAM, epithelial cell adhesion molecule; HER2, human epidermal growth factor receptor 2; MFS, metastasis-free survival; MRD, minimal residual disease; OS, overall survival; PFS, progression-free survival; RT-PCR, reverse transcription polymerase chain reaction.

## References

[1] Ashworth TR A case of cancer in which cells similar to those in tumors were seen in the blood after death. Aust Med J (1869) 14:146–9.

[2] Paget S Distribution of secondary growths in cancer of the breast. Lancet (1889) 1:571 10.1016/S0140-6736(00)49915-0 2673568

[3] Braun S Vogl FD Naume B Janni W Osborne MP Coombes RC A pooled analysis of bone marrow micrometastasis in breast cancer. N Engl J Med (2005) 353(8):793–802. 10.1056/NEJMoa050434 16120859

[4] Banys M Hartkopf AD Krawczyk N Kaiser T Meier-Stiegen F Fehm T Dormancy in breast cancer. Breast Cancer (Dove Med Press) (2012) 4:183–91. 10.2147/BCTT.S26431 24367205 PMC3846810

[5] Pantel K Brakenhoff RH Dissecting the metastatic cascade. Nat Rev Cancer (2004) 4(6):448–56. 10.1038/nrc1370 15170447

[6] Mohme M Riethdorf S Pantel K. Circulating and disseminated tumour cells – mechanisms of immune surveillance and escape. Nat Rev Clin Oncol (2016). 10.1038/nrclinonc.2016.144 27644321

[7] Baccelli I Schneeweiss A Riethdorf S Stenzinger A Schillert A Vogel V Identification of a population of blood circulating tumor cells from breast cancer patients that initiates metastasis in a xenograft assay. Nat Biotechnol (2013) 31(6):539–44. 10.1038/nbt.2576 23609047

[8] Steinert G Scholch S Niemietz T Iwata N Garcia SA Behrens B Immune escape and survival mechanisms in circulating tumor cells of colorectal cancer. Cancer Res (2014) 74(6):1694–704. 10.1158/0008-5472.CAN-13-1885 24599131

[9] Nagahara M Mimori K Kataoka A Ishii H Tanaka F Nakagawa T Correlated expression of CD47 and SIRPA in bone marrow and in peripheral blood predicts recurrence in breast cancer patients. Clin Cancer Res (2010) 16(18):4625–35. 10.1158/1078-0432.CCR-10-0349 20705613

[10] Pantel K Schlimok G Kutter D Schaller G Genz T Wiebecke B Frequent down-regulation of major histocompatibility class I antigen expression on individual micrometastatic carcinoma cells. Cancer Res (1991) 51(17):4712–5. 1873815

[11] Kaplan RN Riba RD Zacharoulis S Bramley AH Vincent L Costa C VEGFR1-positive haematopoietic bone marrow progenitors initiate the pre-metastatic niche. Nature (2005) 438(7069):820–7. 10.1038/nature04186 16341007 PMC2945882

[12] Olkhanud PB Baatar D Bodogai M Hakim F Gress R Anderson RL Breast cancer lung metastasis requires expression of chemokine receptor CCR4 and regulatory T cells. Cancer Res (2009) 69(14):5996–6004. 10.1158/0008-5472.CAN-08-4619 19567680 PMC2743688

[13] Rack B Schindlbeck C Juckstock J Andergassen U Hepp P Zwingers T Circulating tumor cells predict survival in early average-to-high risk breast cancer patients. J Natl Cancer Inst (2014) 106(5). 10.1093/jnci/dju066 24832787 PMC4112925

[14] Bidard FC Belin L Delaloge S Lerebours F Ngo C Reyal F Time-dependent prognostic impact of circulating tumor cells detection in non-metastatic breast cancer: 70-month analysis of the REMAGUS02 study. Int J Breast Cancer (2013) 2013:130470. 10.1155/2013/130470 23762567 PMC3665249

[15] Franken B de Groot MR Mastboom WJ Vermes I van der Palen J Tibbe AG Circulating tumor cells, disease recurrence and survival in newly diagnosed breast cancer. Breast Cancer Res (2012) 14(5):R133. 10.1186/bcr3333 23088337 PMC4053111

[16] Janni WJ Rack B Terstappen LW Pierga JY Taran FA Fehm T Pooled analysis of the prognostic relevance of circulating tumor cells in primary breast cancer. Clin Cancer Res (2016) 22(10):2583–93. 10.1158/1078-0432.CCR-15-1603 26733614

[17] Ignatiadis M Xenidis N Perraki M Apostolaki S Politaki E Kafousi M Different prognostic value of cytokeratin-19 mRNA positive circulating tumor cells according to estrogen receptor and HER2 status in early-stage breast cancer. J Clin Oncol (2007) 25(33):5194–202. 10.1200/JCO.2007.11.7762 17954712

[18] Ignatiadis M Perraki M Apostolaki S Politaki E Xenidis N Kafousi M Molecular detection and prognostic value of circulating cytokeratin-19 messenger RNA-positive and HER2 messenger RNA-positive cells in the peripheral blood of women with early-stage breast cancer. Clin Breast Cancer (2007) 7(11):883–9. 10.3816/CBC.2007.n.054 18269779

[19] Hwang SB Bae JW Lee HY Kim HY. Circulating tumor cells detected by RT-PCR for CK-20 before surgery indicate worse prognostic impact in triple-negative and HER2 subtype breast cancer. J Breast Cancer (2012) 15(1):34–42. 10.4048/jbc.2012.15.1.34 22493626 PMC3318172

[20] Karhade M Hall C Mishra P Anderson A Kuerer H Bedrosian I Circulating tumor cells in non-metastatic triple-negative breast cancer. Breast Cancer Res Treat (2014) 147(2):325–33. 10.1007/s10549-014-3103-7 25164970 PMC4149877

[21] Banys-Paluchowski M Schneck H Blassl C Schultz S Meier-Stiegen F Niederacher D Prognostic relevance of circulating tumor cells in molecular subtypes of breast cancer. Geburtshilfe Frauenheilkd (2015) 75(3):232–7. 10.1055/s-0035-1545788 25914415 PMC4397936

[22] Lim E Metzger-Filho O Winer EP. The natural history of hormone receptor-positive breast cancer. Oncology (Williston Park) (2012) 26(8):688–94. 22957400

[23] Lucci A Hall CS Lodhi AK Bhattacharyya A Anderson AE Xiao L Circulating tumour cells in non-metastatic breast cancer: a prospective study. Lancet Oncol (2012) 13(7):688–95. 10.1016/S1470-2045(12)70209-7 22677156

[24] Molloy TJ Bosma AJ Baumbusch LO Synnestvedt M Borgen E Russnes HG The prognostic significance of tumour cell detection in the peripheral blood versus the bone marrow in 733 early-stage breast cancer patients. Breast Cancer Res (2011) 13(3):R61. 10.1186/bcr2898 21672237 PMC3218950

[25] Kuniyoshi RK de Sousa Gehrke F Alves BC Vilas-Boas V Colo AE Sousa N Gene profiling and circulating tumor cells as biomarker to prognostic of patients with locoregional breast cancer. Tumour Biol (2015) 36(10):8075–83. 10.1007/s13277-015-3529-5 25976504

[26] Fehm T Hoffmann O Aktas B Becker S Solomayer EF Wallwiener D Detection and characterization of circulating tumor cells in blood of primary breast cancer patients by RT-PCR and comparison to status of bone marrow disseminated cells. Breast Cancer Res (2009) 11(4):R59. 10.1186/bcr2349 19664291 PMC2750121

[27] Wulfing P Borchard J Buerger H Heidl S Zanker KS Kiesel L HER2-positive circulating tumor cells indicate poor clinical outcome in stage I to III breast cancer patients. Clin Cancer Res (2006) 12(6):1715–20. 10.1158/1078-0432.CCR-05-2087 16551854

[28] Kasimir-Bauer S Reiter K Aktas B Bittner AK Weber S Keller T Different prognostic value of circulating and disseminated tumor cells in primary breast cancer: influence of bisphosphonate intake? Sci Rep (2016) 6:26355. 10.1038/srep26355 27212060 PMC4876469

[29] Schindlbeck C Andergassen U Hofmann S Juckstock J Jeschke U Sommer H Comparison of circulating tumor cells (CTC) in peripheral blood and disseminated tumor cells in the bone marrow (DTC-BM) of breast cancer patients. J Cancer Res Clin Oncol (2013) 139(6):1055–62. 10.1007/s00432-013-1418-0 23525580 PMC11824800

[30] Hartkopf AD Stefanescu D Wallwiener M Hahn M Becker S Solomayer EF Tumor cell dissemination to the bone marrow and blood is associated with poor outcome in patients with metastatic breast cancer. Breast Cancer Res Treat (2014) 147(2):345–51. 10.1007/s10549-014-3113-5 25151295

[31] Slade MJ Payne R Riethdorf S Ward B Zaidi SA Stebbing J Comparison of bone marrow, disseminated tumour cells and blood-circulating tumour cells in breast cancer patients after primary treatment. Br J Cancer (2009) 100(1):160–6. 10.1038/sj.bjc.6604773 19034279 PMC2634698

[32] Janni W Vogl FD Wiedswang G Synnestvedt M Fehm T Juckstock J Persistence of disseminated tumor cells in the bone marrow of breast cancer patients predicts increased risk for relapse – a European pooled analysis. Clin Cancer Res (2011) 17(9):2967–76. 10.1158/1078-0432.CCR-10-2515 21415211

[33] Janni W Rack B Fasching P Haeberle L Friedl T Tesch H Persistence of circulating tumor cells in high risk early breast cancer patients during follow-up care suggests poor prognosis – results from the adjuvant SUCCESS A trial. 2015 San Antonio Breast Cancer Symposium, S2-03. San Antonio, TX (2015).

[34] Riethdorf S Muller V Zhang L Rau T Loibl S Komor M Detection and HER2 expression of circulating tumor cells: prospective monitoring in breast cancer patients treated in the neoadjuvant GeparQuattro trial. Clin Cancer Res (2010) 16(9):2634–45. 10.1158/1078-0432.CCR-09-2042 20406831

[35] Kasimir-Bauer S Bittner AK Konig L Reiter K Keller T Kimmig R Does primary neoadjuvant systemic therapy eradicate minimal residual disease? Analysis of disseminated and circulating tumor cells before and after therapy. Breast Cancer Res (2016) 18(1):20. 10.1186/s13058-016-0679-3 26868521 PMC4751719

[36] Hall C Karhade M Laubacher B Anderson A Kuerer H DeSynder S Circulating tumor cells after neoadjuvant chemotherapy in stage I-III triple-negative breast cancer. Ann Surg Oncol (2015) 22(Suppl 3):S552–8. 10.1245/s10434-015-4600-6 25968619

[37] Pierga JY Bidard FC Mathiot C Brain E Delaloge S Giachetti S Circulating tumor cell detection predicts early metastatic relapse after neoadjuvant chemotherapy in large operable and locally advanced breast cancer in a phase II randomized trial. Clin Cancer Res (2008) 14(21):7004–10. 10.1158/1078-0432.CCR-08-0030 18980996

[38] Bidard FC Mathiot C Delaloge S Brain E Giachetti S de Cremoux P Single circulating tumor cell detection and overall survival in nonmetastatic breast cancer. Ann Oncol (2010) 21(4):729–33. 10.1093/annonc/mdp391 19850639

[39] Cortazar P Zhang L Untch M Mehta K Costantino JP Wolmark N Pathological complete response and long-term clinical benefit in breast cancer: the CTNeoBC pooled analysis. Lancet (2014) 384(9938):164–72. 10.1016/S0140-6736(13)62422-8 24529560

[40] Xenidis N Perraki M Apostolaki S Agelaki S Kalbakis K Vardakis N Differential effect of adjuvant taxane-based and taxane-free chemotherapy regimens on the CK-19 mRNA-positive circulating tumour cells in patients with early breast cancer. Br J Cancer (2013) 108(3):549–56. 10.1038/bjc.2012.597 23329233 PMC3593552

[41] Banys M Muller V Melcher C Aktas B Kasimir-Bauer S Hagenbeck C Circulating tumor cells in breast cancer. Clin Chim Acta (2013) 423:39–45. 10.1016/j.cca.2013.03.029 23623805

[42] Fehm T Krawczyk N Solomayer EF Becker-Pergola G Durr-Storzer S Neubauer H ERalpha-status of disseminated tumour cells in bone marrow of primary breast cancer patients. Breast Cancer Res (2008) 10(5):R76. 10.1186/bcr2143 18793387 PMC2614509

[43] Krawczyk N Banys M Neubauer H Solomayer EF Gall C Hahn M HER2 status on persistent disseminated tumor cells after adjuvant therapy may differ from initial HER2 status on primary tumor. Anticancer Res (2009) 29(10):4019–24. 19846945

[44] Rack B Juckstock J Gunthner-Biller M Andergassen U Neugebauer J Hepp P Trastuzumab clears HER2/neu-positive isolated tumor cells from bone marrow in primary breast cancer patients. Arch Gynecol Obstet (2012) 285(2):485–92. 10.1007/s00404-011-1954-2 21717141

[45] Ignatiadis M Rack B Rothe F Riethdorf S Decraene C Bonnefoi H Liquid biopsy-based clinical research in early breast cancer: the EORTC 90091-10093 Treat CTC Trial. Eur J Cancer (2016) 63:97–104. 10.1016/j.ejca.2016.04.024 27289552

[46] Cristofanilli M Hayes DF Budd GT Ellis MJ Stopeck A Reuben JM Circulating tumor cells: a novel prognostic factor for newly diagnosed metastatic breast cancer. J Clin Oncol (2005) 23(7):1420–30. 10.1200/JCO.2005.08.140 15735118

[47] Cristofanilli M Budd GT Ellis MJ Stopeck A Matera J Miller MC Circulating tumor cells, disease progression, and survival in metastatic breast cancer. N Engl J Med (2004) 351(8):781–91. 10.1056/NEJMoa040766 15317891

[48] Budd GT Cristofanilli M Ellis MJ Stopeck A Borden E Miller MC Circulating tumor cells versus imaging – predicting overall survival in metastatic breast cancer. Clin Cancer Res (2006) 12(21):6403–9. 10.1158/1078-0432.CCR-05-1769 17085652

[49] Hayes DF Cristofanilli M Budd GT Ellis MJ Stopeck A Miller MC Circulating tumor cells at each follow-up time point during therapy of metastatic breast cancer patients predict progression-free and overall survival. Clin Cancer Res (2006) 12(14 Pt 1):4218–24. 10.1158/1078-0432.CCR-05-2821 16857794

[50] Giuliano M Giordano A Jackson S Hess KR De Giorgi U Mego M Circulating tumor cells as prognostic and predictive markers in metastatic breast cancer patients receiving first-line systemic treatment. Breast Cancer Res (2011) 13(3):R67. 10.1186/bcr2907 21699723 PMC3218956

[51] Giordano A Giuliano M De Laurentiis M Arpino G Jackson S Handy BC Circulating tumor cells in immunohistochemical subtypes of metastatic breast cancer: lack of prediction in HER2-positive disease treated with targeted therapy. Ann Oncol (2012) 23(5):1144–50. 10.1093/annonc/mdr434 21965473

[52] Wallwiener M Hartkopf AD Baccelli I Riethdorf S Schott S Pantel K The prognostic impact of circulating tumor cells in subtypes of metastatic breast cancer. Breast Cancer Res Treat (2013) 137(2):503–10. 10.1007/s10549-012-2382-0 23271327

[53] Bidard FC Peeters DJ Fehm T Nole F Gisbert-Criado R Mavroudis D Clinical validity of circulating tumour cells in patients with metastatic breast cancer: a pooled analysis of individual patient data. Lancet Oncol (2014) 15(4):406–14. 10.1016/S1470-2045(14)70069-5 24636208

[54] Smerage JB Barlow WE Hortobagyi GN Winer EP Leyland-Jones B Srkalovic G Circulating tumor cells and response to chemotherapy in metastatic breast cancer: SWOG S0500. J Clin Oncol (2014) 32(31):3483–9. 10.1200/JCO.2014.56.2561 24888818 PMC4209100

[55] Pierga JY Hajage D Bachelot T Delaloge S Brain E Campone M High independent prognostic and predictive value of circulating tumor cells compared with serum tumor markers in a large prospective trial in first-line chemotherapy for metastatic breast cancer patients. Ann Oncol (2012) 23(3):618–24. 10.1093/annonc/mdr263 21642515

[56] Müller V Riethdorf S Rack B Janni W Fasching PA Solomayer E Prognostic impact of circulating tumor cells assessed with the CellSearch System and AdnaTest Breast in metastatic breast cancer patients: the DETECT study. Breast Cancer Res (2012) 14(4):R118 10.1186/bcr3243 22894854 PMC3680931

[57] Nakamura S Yagata H Ohno S Yamaguchi H Iwata H Tsunoda N Multi-center study evaluating circulating tumor cells as a surrogate for response to treatment and overall survival in metastatic breast cancer. Breast Cancer (2010) 17(3):199–204. 10.1007/s12282-009-0139-3 19649686

[58] Liu MC Shields PG Warren RD Cohen P Wilkinson M Ottaviano YL Circulating tumor cells: a useful predictor of treatment efficacy in metastatic breast cancer. J Clin Oncol (2009) 27(31):5153–9. 10.1200/JCO.2008.20.6664 19752342 PMC4879719

[59] Tewes M Aktas B Welt A Mueller S Hauch S Kimmig R Molecular profiling and predictive value of circulating tumor cells in patients with metastatic breast cancer: an option for monitoring response to breast cancer related therapies. Breast Cancer Res Treat (2009) 115(3):581–90. 10.1007/s10549-008-0143-x 18679793

[60] Bidard FC Vincent-Salomon A Sigal-Zafrani B Dieras V Mathiot C Mignot L Prognosis of women with stage IV breast cancer depends on detection of circulating tumor cells rather than disseminated tumor cells. Ann Oncol (2008) 19(3):496–500. 10.1093/annonc/mdm507 18187488

[61] Nole F Munzone E Zorzino L Minchella I Salvatici M Botteri E Variation of circulating tumor cell levels during treatment of metastatic breast cancer: prognostic and therapeutic implications. Ann Oncol (2008) 19(5):891–7. 10.1093/annonc/mdm558 18056915

[62] Kalinsky K Mayer JA Xu X Pham T Wong KL Villarin E Correlation of hormone receptor status between circulating tumor cells, primary tumor, and metastasis in breast cancer patients. Clin Transl Oncol (2015) 17(7):539–46. 10.1007/s12094-015-1275-1 25613123 PMC4497875

[63] Babayan A Hannemann J Spotter J Muller V Pantel K Joosse SA. Heterogeneity of estrogen receptor expression in circulating tumor cells from metastatic breast cancer patients. PLoS One (2013) 8(9):e75038. 10.1371/journal.pone.0075038 24058649 PMC3776726

[64] Pestrin M Bessi S Galardi F Truglia M Biggeri A Biagioni C Correlation of HER2 status between primary tumors and corresponding circulating tumor cells in advanced breast cancer patients. Breast Cancer Res Treat (2009) 118(3):523–30. 10.1007/s10549-009-0461-7 19597704

[65] Wallwiener M Hartkopf AD Riethdorf S Nees J Sprick MR Schonfisch B The impact of HER2 phenotype of circulating tumor cells in metastatic breast cancer: a retrospective study in 107 patients. BMC Cancer (2015) 15:403. 10.1186/s12885-015-1423-6 25972110 PMC4435916

[66] Fehm T Muller V Aktas B Janni W Schneeweiss A Stickeler E HER2 status of circulating tumor cells in patients with metastatic breast cancer: a prospective, multicenter trial. Breast Cancer Res Treat (2010) 124(2):403–12. 10.1007/s10549-010-1163-x 20859679

[67] Pantel K Alix-Panabieres C. Real-time liquid biopsy in cancer patients: fact or fiction? Cancer Res (2013) 73(21):6384–8. 10.1158/0008-5472.CAN-13-2030 24145355

[68] Bernhard H Neudorfer J Gebhard K Conrad H Hermann C Nahrig J Adoptive transfer of autologous, HER2-specific, cytotoxic T lymphocytes for the treatment of HER2-overexpressing breast cancer. Cancer Immunol Immunother (2008) 57(2):271–80. 10.1007/s00262-007-0355-7 17646988 PMC11030865

[69] Bozionellou V Mavroudis D Perraki M Papadopoulos S Apostolaki S Stathopoulos E Trastuzumab administration can effectively target chemotherapy-resistant cytokeratin-19 messenger RNA-positive tumor cells in the peripheral blood and bone marrow of patients with breast cancer. Clin Cancer Res (2004) 10(24):8185–94. 10.1158/1078-0432.CCR-03-0094 15623593

[70] Hayashi N Nakamura S Tokuda Y Shimoda Y Yagata H Yoshida A Prognostic value of HER2-positive circulating tumor cells in patients with metastatic breast cancer. Int J Clin Oncol (2012) 17(2):96–104. 10.1007/s10147-011-0260-0 21671160 PMC3860324

[71] Beije N Onstenk W Kraan J Sieuwerts AM Hamberg P Dirix LY Prognostic impact of HER2 and ER status of circulating tumor cells in metastatic breast cancer patients with a HER2-negative primary tumor. Neoplasia (2016) 18(11):647–53. 10.1016/j.neo.2016.08.007 27764697 PMC5071539

[72] De Luca F Rotunno G Salvianti F Galardi F Pestrin M Gabellini S Mutational analysis of single circulating tumor cells by next generation sequencing in metastatic breast cancer. Oncotarget (2016) 7(18):26107–19. 10.18632/oncotarget.8431 27034166 PMC5041968

[73] Heitzer E Auer M Gasch C Pichler M Ulz P Hoffmann EM Complex tumor genomes inferred from single circulating tumor cells by array-CGH and next-generation sequencing. Cancer Res (2013) 73(10):2965–75. 10.1158/0008-5472.CAN-12-4140 23471846

[74] Bidard FC Fehm T Ignatiadis M Smerage JB Alix-Panabieres C Janni W Clinical application of circulating tumor cells in breast cancer: overview of the current interventional trials. Cancer Metastasis Rev (2013) 32(1–2):179–88. 10.1007/s10555-012-9398-0 23129208 PMC3655223

